# Supplementary material for: Functional genomics screens reveal a role for TBC1D24 and SV2B in antibody-dependent enhancement of dengue virus infection
Source: bioRxiv. 2024 Apr 27:2024.04.26.591029. Preprint. [Version 1] doi: 10.1101/2024.04.26.591029 (PMC11071485; doi:10.1101/2024.04.26.591029)
Supplement: Supplement 5 [file media-5.pdf]

Figure S1

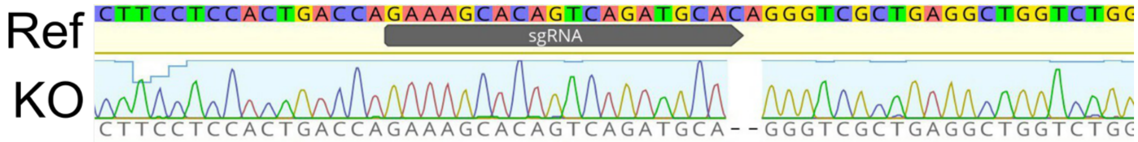

Fig S1: Genotyping of K562 FcgRIIa KO clone.

Sanger sequencing of locus targeted by sgRNA in the K562 FcgRIIa KO clonal line. Traces were aligned to WT reference sequence (“Ref”) to identify the indicated deletion.
