# Supplementary material for: Functional genomics screens reveal a role for TBC1D24 and SV2B in antibody-dependent enhancement of dengue virus infection
Source: bioRxiv. 2024 Apr 27:2024.04.26.591029. Preprint. [Version 1] doi: 10.1101/2024.04.26.591029 (PMC11071485; doi:10.1101/2024.04.26.591029)
Supplement: Supplement 7 [file media-7.pdf]

Figure S3

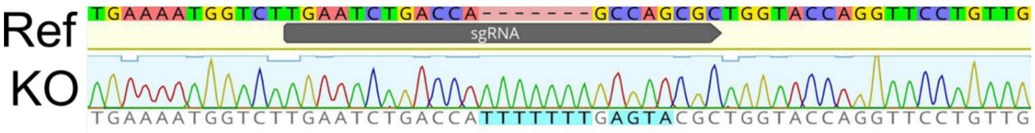

Fig S3: Genotyping of K562 SV2B KO clone.
Sanger sequencing of locus targeted by sgRNA in K562 SV2B KO clonal cell line. Traces were aligned to WT reference sequence (“Ref”) to identify a 7 bp insertion and 4 bp missense mutation.
