# Supplementary material for: Functional genomics screens reveal a role for TBC1D24 and SV2B in antibody-dependent enhancement of dengue virus infection
Source: bioRxiv. 2024 Apr 27:2024.04.26.591029. Preprint. [Version 1] doi: 10.1101/2024.04.26.591029 (PMC11071485; doi:10.1101/2024.04.26.591029)
Supplement: Supplement 9 [file media-9.pdf]

Figure S5

Clone	KO Score
KO_1	92
KO_2	nd
KO_3	97
KO_4	nd

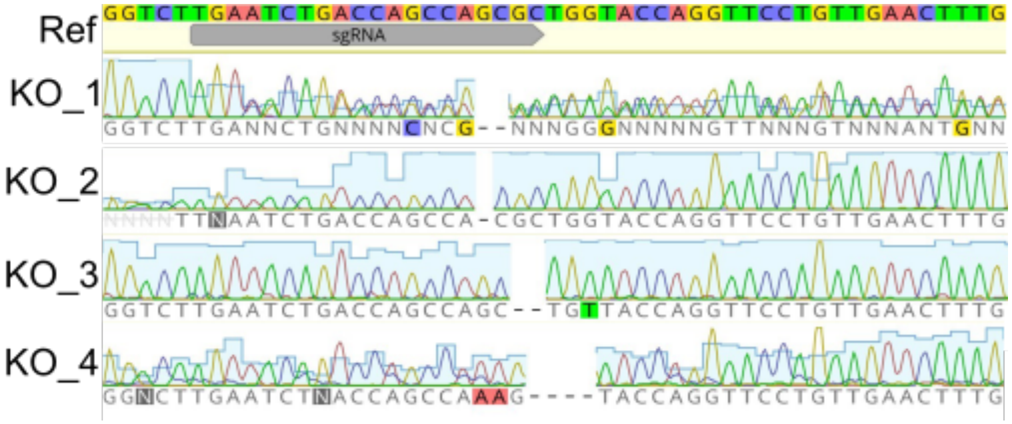

Fig S5: Genotyping of K562-DCSIGN SV2B KO clones.
(Right) Sanger sequencing of locus targeted by sgRNA in the K562-DCSIGN SV2B KO clones. Traces were aligned to WT reference sequence and heterogeneous mutations deconvoluted using Inference of CRISPR Edits (ICE; https://ice.synthego.com/#/). (Left) KO scores as determined by ICE; nd = not determined.
