# Supplementary material for: Functional genomics screens reveal a role for TBC1D24 and SV2B in antibody-dependent enhancement of dengue virus infection
Source: bioRxiv. 2024 Apr 27:2024.04.26.591029. Preprint. [Version 1] doi: 10.1101/2024.04.26.591029 (PMC11071485; doi:10.1101/2024.04.26.591029)
Supplement: Supplement 10 [file media-10.pdf]

Figure S6

Clone	KO Score
KO_1	100
KO_2	92
KO_3	100
KO_4	93
KO_5	100
KO_6	100

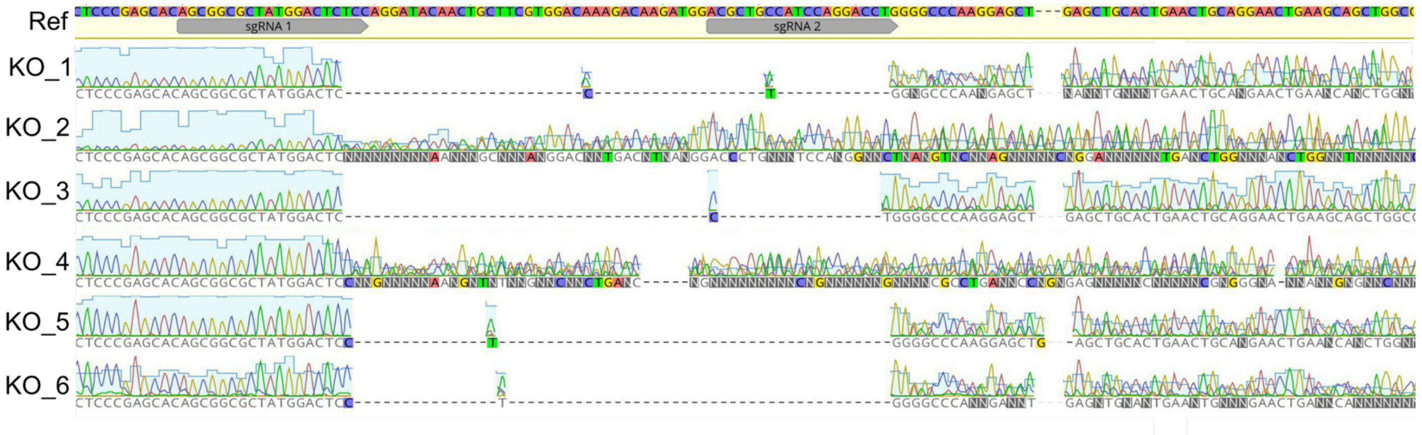

Fig S6: Genotyping of K562-DCSIGN TBC1D24 KO clones.
(Right) Sanger sequencing of locus targeted by gRNA in K562-DCSIGN TBC1D24 KO clones. Traces were aligned to WT reference sequence and heterogeneous mutations deconvoluted using ICE (https://ice.synthego.com/#/). (Left) KO scores as determined by ICE.
