# Supplementary material for: Functional genomics screens reveal a role for TBC1D24 and SV2B in antibody-dependent enhancement of dengue virus infection
Source: bioRxiv. 2024 Apr 27:2024.04.26.591029. Preprint. [Version 1] doi: 10.1101/2024.04.26.591029 (PMC11071485; doi:10.1101/2024.04.26.591029)
Supplement: Supplement 11 [file media-11.pdf]

Figure S7

Clone	KO Score
KO_1	100
KO_2	100
KO_3	100
KO_4	100
KO_5	95
KO_6	100
KO_7	100
KO_8	100
KO_9	96
KO_10	100
KO_11	100

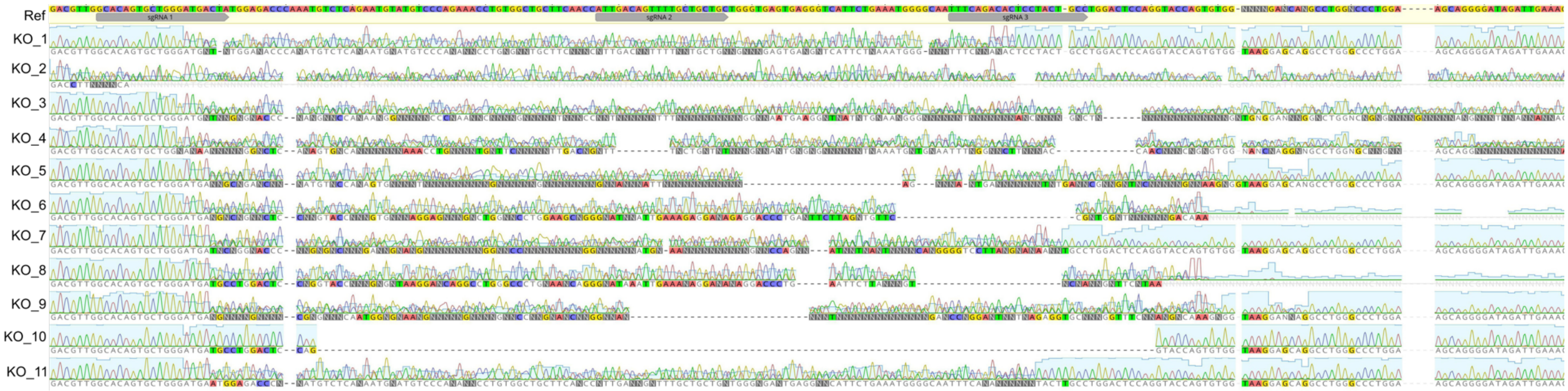

Fig S7: Genotyping of K562-DCSIGN FcgRIIa KO clones
(Right) Sanger sequencing of locus targeted by gRNA K562-DCSIGN FcgRIIa KO clones. Traces were aligned to WT reference sequence and heterogeneous mutations deconvoluted using ICE (https://ice.synthego.com/#/). (Left) KO scores as determined by ICE.
