# Supplementary material for: Functional genomics screens reveal a role for TBC1D24 and SV2B in antibody-dependent enhancement of dengue virus infection
Source: bioRxiv. 2024 Apr 27:2024.04.26.591029. Preprint. [Version 1] doi: 10.1101/2024.04.26.591029 (PMC11071485; doi:10.1101/2024.04.26.591029)
Supplement: Supplement 13 [file media-13.pdf]

Figure S9

Clone	KO Score
KO_1	100
KO_2	100
KO_3	100
KO_4	100

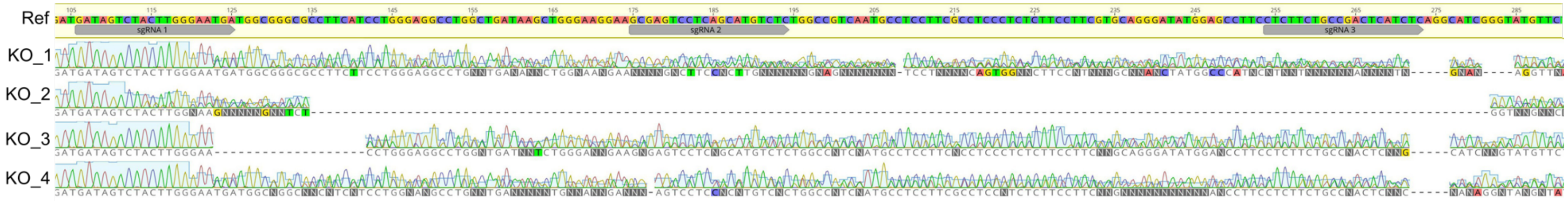

Fig S9: Genotyping of U937 SV2B KO clones

(Right) Sanger sequencing of locus targeted by gRNA in U937 SV2B KO clones. Traces were aligned to WT reference sequence and heterogeneous mutations deconvoluted using ICE (https://ice.synthego.com/#/). (Left) KO scores as determined by ICE.
